# Seroconversion in asymptomatic COVID-19 pediatric patients with rheumatic diseases of one tertiary referral hospital

**DOI:** 10.1016/j.clinsp.2022.100110

**Published:** 2022-09-12

**Authors:** Juliana R. Simon, Maria F.B. Pereira, Heloisa H. Marques, Adriana M. Elias, Neusa K. Sakita, Juliana C.O.A. Ferreira, Alexander Roberto Precioso, Sandra J.F.E. Grisi, Ana Paula S. Ferrer, Vera Bain, Clovis A. Silva, Lúcia M.A. Campos

**Affiliations:** a Pediatric Rheumatology Unit, Instituto da Criança e do Adolescente, Hospital das Clínicas, Faculdade de Medicina da Universidade de São Paulo (HCFMUSP), São Paulo, SP, Brazil; b Pediatric Infectology Unit, Instituto da Criança e do Adolescente, Hospital das Clínicas, Faculdade de Medicina da Universidade de São Paulo (HCFMUSP), São Paulo, SP, Brazil; c Rheumatology Division, Hospital das Clínicas, Faculdade de Medicina da Universidade de São Paulo (HCFMUSP), São Paulo, SP, Brazil; d Clinical Research Center, Instituto da Criança e do Adolescente, Hospital das Clínicas, Faculdade de Medicina da Universidade de São Paulo (HCFMUSP), São Paulo, SP, Brazil; e Department of Infectious and Parasitic Diseases, Faculdade de Medicina da Universidade de São Paulo (FMUSP), São Paulo, SP, Brazil; f Division of Clinical Trials and Pharmacovigilance, Instituto Butantan, São Paulo, SP, Brazil; g Instituto da Criança e do Adolescente, Hospital das Clínicas, Faculdade de Medicina da Universidade de São Paulo (HCFMUSP), São Paulo, SP, Brazil

**Keywords:** COVID-19 testing, Rheumatic diseases, Immunosuppressive treatment, SARS-CoV-2, Serology, Children

## Abstract

**Objectives:**

To evaluate seroconverted asymptomatic COVID-19 in pediatric Autoimmune Rheumatic Diseases (ARDs) patients and to identify the risk factors related to contagion.

**Methods:**

A cross-sectional study was conducted in March 2021, before vaccination of children and adolescents in Brazil, including 77 pediatric ARDs patients, followed at a tertiary hospital and 45 healthy controls, all of them without a previous diagnosis of COVID-19. Data was obtained by a questionnaire with demographic data, symptoms compatible with COVID-19 over the previous year, and contact with people with confirmed COVID-19. Patient's medical records were reviewed to access data regarding disease and current medications. A qualitative immunochromatographic SARS-CoV-2 test was performed on all participants.

**Results:**

Patients and controls were similar in terms of female gender (70.1% vs. 57.8%, p = 0.173), age (14 vs. 13 years, p = 0.269) and SARS-CoV-2 positive serology (22% vs. 15.5%, p = 0.481). 80.5% of rheumatic patients were in use of immunosuppressive drugs: 27.3% of them used corticosteroids (33.3% in high doses), and 7.8% on immunobiologicals. No statistical differences were found between positive (n = 17) and negative serology (n = 60) patients regarding demographic/socioeconomic data, contact with people with confirmed COVID-19, use and number of immunosuppressive drugs, use and dose of corticosteroids, use of hydroxychloroquine and immunobiological drugs (p > 0.05).

**Conclusions:**

Pediatric rheumatic disease patients were infected at the same rate as healthy ones. Neither the underlying pathology nor its immunosuppressive treatment seemed to interfere with contagion risk.

## Highlights

• The study evaluated seroconverted asymptomatic COVID-19 in pediatric Autoimmune Rheumatic Diseases (ARDs) patients.
• Pediatric rheumatic disease patients were infected at the same rate as healthy ones.
• Neither the underlying pathology nor its immunosuppressive treatment seemed to interfere with contagion risk.

## Introduction

The Coronavirus Disease-19 (COVID-19) is caused by the virus named Severe Acute Respiratory Syndrome Coronavirus-2 (SARS-CoV-2), initially described in the province of Wuhan, China, in December 2019.^1^ It is recognized as one of the biggest pandemics the world has ever seen and caused more than 670 thousand deaths only in Brazil and more than 6.4 million deaths worldwide until August 2022.^2^

The disease is responsible for mild to moderate presentation in approximately 80% of people, and 20% of adults progress to severe disease and require hospitalization, with a mortality rate ranging from 0.5% to 4%.^3^, ^4^, ^5^ Asymptomatic infections are described, but the percentage is difficult to estimate since these patients often do not seek medical attention. A systematic review showed wide variation between different studies, ranging from 1% to 50% of cases.^6^

As the virus spread around the world throughout 2020, questions concerning the behavior of the infection in immunocompromised patients have arisen. Several studies and cohorts with adult populations were carried out over the last year showing that patients with pediatric Autoimmune Rheumatic Diseases (ARDs) were more likely to be infected and to develop more severe forms of COVID-19,^6^, ^7^, ^8^, ^9^, ^10^, ^11^ since they present immunosuppressive mechanisms inherent to the disease itself and to its treatment.^7^ In fact, the use of corticosteroids and Disease-Modifying Antirheumatic Drugs (DMARDs) were identified as risk factors for more aggressive diseases.^4^^,^^8^, ^9^, ^10^, ^11^

Since the beginning of the pandemic, some studies suggested that healthy children could have a more indolent course of COVID-19.^12^^,^^13^ Immunosuppressed children have not been as extensively studied as adults in the same condition. Few studies have been published showing that children with chronic immunosuppressive diseases had symptomatic COVID-19 at the same rate and severity as healthy controls.^14^, ^15^, ^16^ The present group has already published our experience with pediatric patients with ARDs and symptomatic COVID-19, demonstrating that it affected just 2% of the population, with mild to moderate manifestations.^17^ Based on previous studies in healthy children and in pediatric immunosuppressed populations.^12^, ^13^, ^14^, ^15^, ^16^, ^17^ The authors hypothesized that children with ARDs, regardless of the use of immunosuppressors, are infected by COVID-19 similarly to the healthy ones, considering that demographic characteristics may be the more determinant factor in contagion.

To our knowledge, there are no studies on asymptomatic or oligosymptomatic COVID-19 in children with ARDs and there are several unanswered questions surrounding this subject.

Therefore, the objectives of the present study are to evaluate seroconversion of asymptomatic SARS-CoV-2 infection in pediatric ARDs patients and healthy controls, and to identify the risk factors related to contagion in this population.

## Methods

### Study population

This is a cross-sectional study performed with patients followed at the Pediatric Rheumatology Unit of a Brazilian tertiary hospital, located in the city of São Paulo, conducted between March 1^st^ to March 31^st^, 2021, before starting the vaccination of children and adolescents in Brazil. Ninety-two patients with ARDs who attended the outpatient clinic or were in the hospital for other procedures in this period were invited to participate. Four of them declined to participate in the study, totaling 88 patients. It included patients with Juvenile Idiopathic Arthritis (JIA), Juvenile Systemic Lupus Erythematosus (JSLE), Juvenile Dermatomyositis (JDM), scleroderma, Mixed Connective Tissue Disease (MCTD), vasculitis (Behçet disease and IgA Vasculitis) and IgG4 disease. The authors also invited 56 healthy children and adolescents to the control group, who were patients' family members or patients´ close friends and hospital staffs' children; they were invited to participate in the study by telephone or when they accompanied their relatives to outpatient consultations, and ten of them refused to participate in the study, totalizing 46 controls.

The study included subjects between one to 18 years old who did not have a previous diagnosis of COVID-19 by Reverse Transcription Polymerase Chain Reaction (RT-PCR) or serology over the last year and did not present signs or symptoms of COVID-19 strong enough to alert them for the possibility of the disease or to led them to seek for medical attention, such as persistent fever, flu-like syndrome, prostration, dyspnea, intense cough, persistent chest pain or pressure, severe myalgia, abdominal pain, diarrhea, mental confusion, anosmia or dysgeusia.^18^, ^19^, ^20^

Children under one year of age were excluded due to the possibility of having circulating maternal antibodies, and above 19 years old, who are already considered adults according to the World Health Organization (WHO) criteria.^21^ People who had shown warning signs for COVID-19 or who had been diagnosed with the disease in the previous year were excluded, as well as subjects who had signs of active infection on the day of sample collection. Patients who received Rituximab (RTX) or Intravenous Immunoglobulin (IVIG) in the six months prior to the study were also excluded since these medications could interfere with adequate antibody identification.^22^, ^23^, ^24^

All participant's guardians and the subjects above nine years old signed the consent form and assent form, respectively. The study was approved by the National Research and Ethics Committee (approval number 4.558.660).

### Questionnaire

Patients and controls answered a questionnaire with demographic data (age, gender, family income, years of maternal education, number of inhabitants in the residence), previous pathologies, symptoms compatible with COVID-19 over the past 12 months and date of occurrence, contact with people with confirmed COVID-19 (as well as the place where it occurred), and contact with other immunosuppressed people in the residence. Patient's medical records were reviewed to obtain data regarding the disease, current medications, their doses (hydroxychloroquine and immunosuppressants agents, such as corticosteroids, DMARDS, cytotoxic and immunomodulatory agents and biological medications), and whether they received RTX or IVIG in the last six months.

### Laboratory analysis

A sample of 3 to 5 mL of peripheral blood was collected from each enrolled participant. The sample was centrifuged and kept under refrigeration (2° to 8°C) for a maximum period of three days. An immunochromatographic qualitative serological test (Wondfo®), which analyzes total (IgG and IgM) antibodies for SARS-CoV-2, was performed for all study subjects, with a sensitivity of 86.43% (95% CI 82.41% ∼ 89.58%) and specificity of 99.57% (95% CI 97.63% ∼ 99,92%).^25^, ^26^, ^27^ The test was considered valid if a colored test band appeared in the control region (C) and positive if a colored line appeared in the test region (T) within 15 minutes of starting the reaction.^26^

### Statistic analysis

Data was recorded in the Microsoft Excel spreadsheet program and analyzed using MedCalc software version 17.8.6 and GraphPad StatMate version 1.01. Categorical variables are presented as frequencies and percentages. Fisher's exact test or Pearson's Chi-Square test were used to comparing these variables. The results of continuous variables were expressed as median (range) or mean (±standard deviation) and compared by Mann-Whitney and Student's *t*-tests, as appropriate; p-values below 0.05 were considered significant.

## Results

134 subjects were eligible to participate in the study, comprising 88 patients and 46 controls. Among patients, seven were excluded due to diagnosis of COVID-19 over the past 12 months and four patients for receiving RTX or IVIG in the past six months, resulting in 77 enrolled patients. In the control group, only one child was excluded for a confirmed previous diagnosis of COVID-19. The final control group consisted of 18 (40%) relatives of employees, 26 (57.8%) relatives, and 1 close friend of the participating patients.

The patients’ group included 31 (40.2%) JIA patients, 25 (32.4%) JSLE, 11 (14.3%) JDM, six patients with vasculitis, two with SS, one MCTD, and one IgG4 disease. For statistical convenience, the last four groups of patients were joined into a single group called Other Pathologies (OP) and represented 13% (10/77) of all patients.

Serology was positive in 17 (22%) patients and 7 (15.5%) controls, without a difference between the groups (p = 0.481). Groups were comparable in female gender (70.1% vs. 57.8%, p = 0.173) and median current age (14 vs. 13 years, p = 0.269). Regarding socioeconomic data, the patient's median family income was significantly lower compared to controls (2.5 vs. 5 minimum wages, p < 0.001), as well as lower median years of maternal education (11 vs. 14 years, p = 0.002), and lower contact with other immunocompromised people at residence (13% vs. 31.1%, p = 0.019) (Table 1). No statistical differences were seen between the groups related to the number of inhabitants in the residence and frequency of contact with COVID-19. These contacts occurred most frequently in their own residences in both patients and controls (76.5% vs. 88.2%, p = 0.656) (Table 1).

**Table 1:** Demographic and socioeconomic data and serological test in pediatric rheumatic patients *vs*. controls.

Variables	Patients (n = 77)	Controls (n = 45)	p-value
Female gender	54 (70.1)	26 (57.8)	0.173
Current age (years)	14 (4‒19)	13 (10‒17)	0.269
Family income (minimum wages)	2.5 (0‒19)	5 (1‒35)	< 0.001
Number of inhabitants in the residence	4 (2‒8)	4 (2‒8)	0.335
Maternal education (years)	11 (3‒17)	14 (3‒17)	0.002
Contact with SARS-CoV-2	17 (22.0)	17 (37.8)	0.093
Contact with COVID-19 at home	13/17 (76.5)	15/17 (88.2)	0.656
Contact with immunocompromised people	10 (13.0)	14 (31.1)	0.019
Positive serology	17 (22.0)	7 (15.5)	0.481

Results presented in frequency (%) and median (range). p-values < 0.05 were considered as significant. SARS-CoV-2, Severe Acute Respiratory Syndrome Coronavirus 2; COVID-19, Disease of Coronavirus-19.

Among patients with ARDs, 10/77 (13%) weren't receiving treatment for baseline disease; 62/77 (80.5%) were in use of immunosuppressive drugs (biological and non-biological), 38.7% (24/62) of them were taking a single immunosuppressive agent, 51.6% (32/62) two agents and 9.6% (6/62) three or more immunosuppressive drugs. Regarding the use of corticosteroids, 21/77 (27.3%) patients were using corticosteroids at the time of collection, and 33.3% of them (7/21) were using it in high doses (≥ 20 mg/day or ≥ 1 mg/kg/day). Among the immunosuppressive agents used, 26 patients were using Methotrexate, nine Leflunomide, seven Azathioprine, four cyclophosphamide, three Cyclosporine, two Sulfasalazine, and one Tacrolimus. Six out of 77 (7.8%) patients were in the use of immunobiological (adalimumab in three patients, etanercept in two, and tocilizumab in one). Hydroxychloroquine was in use by 30/77 (39%) of all patients.

Patients were divided according to serology results: 17 (22%) were positive and 60 (77.9%) were negative (Table 2). These groups were compared regarding demographic and socioeconomic data, and no statistical differences were found in gender, age, family income, number of inhabitants in the residence, and maternal education, as well as in relation to contact with people with confirmed COVID-19 or with other immunocompromised people. Concerning treatment, the groups were similar in terms of the use of hydroxychloroquine and the use and number of immunosuppressive drugs (biological and non-biological). Further analysis showed that the groups were also similar regarding the use and dose of corticosteroids and use of immunobiological drugs (p>0.05). No statistical significance was found between groups with respect to the distribution of different chronic conditions studied (Table 2).

**Table 2:** Demographic and socioeconomic data, use of immunosuppressive drugs and underlying pathology in pediatric rheumatic patients according to serological test results.

	Positive serology(n = 17)	Negative serology(n = 60)	p-value
**Female gender**	13 (76.5)	41 (68.3)	0.764
**Current age (years)**	15 (4‒18)	14 (4‒19)	0.888
**Family income (minimum wages)**	2 (0‒5.5)	2.5 (0.3‒19)	0.210
**Number of inhabitants in the residence**	4 (2‒7)	4 (2‒8)	0.589
**Maternal education (years)**	11 (3‒12)	11 (5‒17)	0.076
**Contact with SARS-CoV-2**	5 (29.4)	12 (20)	0.508
**Contact with immunocompromised people**	3 (17.6)	7 (11.6)	0.682
**Use of hydroxychloroquine**	8 (47.0)	22 (36.6)	0.574
**Use of immunosuppressors**	15 (88.2)	47 (78.3)	0.298
**Number of immunosuppressors**			0.468
n = 1	8 (33.4)	16 (66.6)	
n = 2	6 (18.7)	26 (81.3)	
n = 3 or +	1 (16.7)	5 (83.3)	
**Use of corticosteroids**	3 (17.6)	18 (30)	0.373
**Dose of corticosteroids**			
Median dose (mg/kg/dia)	0.55 (0.15‒0.68)	0.21 (0.05‒2.0)	0.525
High dose (≥ 20 mg/day or ≥ 1 mg/kg/day)	2 (11.6)	5 (8.3)	0.646
**Use of immunobiological drugs**	1 (5.8)	5 (8.3)	1.000
**Pathology**			0.564
JIA	6 (19.4)	25 (80.6)	
JSLE	8 (32)	17 (68)	
JDM	3 (27.3)	8 (72.7)	
Others	0	10 (100)	NA

Results presented in frequency (%) and median (range). p-values < 0.05 were considered as significant. SARS-CoV-2. Severe Acute Respiratory Syndrome Coronavirus 2, JIA, Juvenile Idiopathic Arthritis, JSLE, Juvenile Systemic Lupus Erythematosus; JDM, Juvenile dermatomyositis; NA, Not Applicable.

## Discussion

As hypothesized, the present study demonstrated that children with rheumatic diseases without warning signs for COVID-19 became infected at the same rate as the healthy population, regardless of their immune status or treatment.

The impact of the SARS-CoV-2 pandemic on rheumatic populations is a point of great concern due to their immune status. In fact, studies that analyzed the virus epidemiology and its risk factors in adult rheumatic patients showed that this population presented an increased risk of SARS-CoV-2 infection, especially if they used DMARDs.^7^^,^^8^^,^^10^ Additionally they are also more likely to have hospitalizations for COVID-19, especially those using glucocorticoids.^7^^,^^10^^,^^11^

However, immunosuppressed pediatric populations seem to present a milder outcome. In fact, a study with 113 children and adolescents with kidney diseases with confirmed COVID-19^14^ showed that children, even when using immunosuppressants, did not present a higher incidence of severe cases of the disease. In pediatric ARDs, a previous study carried out by the Pediatric Rheumatology group demonstrated that symptomatic COVID-19 was found in just 2% of the present cases, with the predominance of mild to moderate manifestations and none of them presented flare of their underlying pathology.^17^

Moreover, an American study conducted in a single center in Pennsylvania, investigated the seroprevalence of SARS-CoV-2 in 485 immunocompromised children with various chronic conditions, without systematic evaluation of demographic data or therapeutic characteristics and without analyses of asymptomatic cases, and found that 1% of infection in this population.^15^ Although the present study has found a higher frequency of SARS-CoV-2 infection (22%), the present results are in agreement with the serological survey carried out in the city of São Paulo in 2020.^16^ This survey used the same immunochromatographic test as used herein, and observed a prevalence of 17.6% of positive serology in the general pediatric population. As the authors previously assumed, the present study did not find differences in the positivity of serology for SARS-CoV-2 in asymptomatic children and adolescents, has been similar in those with rheumatic diseases as well as in healthy controls, demonstrating that, unlike adults,^4^^,^^8^^,^^11^^,^^28^ indeed, pediatric populations tend to have fewer symptoms of COVID-19^12^^,^^13^ even if they have immunosuppressive diseases or if in use of immunosuppressive drugs (biological or non-biological).^14^^,^^15^^,^^17^

In the serological survey carried out in the city of São Paulo throughout 2020, it was observed that adults were more likely to become infected when they had lower income and education, and while living in houses with five or more inhabitants.^29^ In the present study, the authors analyzed these data in patients and controls, and, even though the groups had some significant socio-economical differences, such as family income and mothers' education, there was still no difference in the frequency of asymptomatic or oligosymptomatic cases. It is important to emphasize that in most patients and controls the contact with a confirmed case of COVID-19 occurred within their own residences since their relatives still leave for work. Regarding contact with immunocompromised people, as was expected, it was higher in the control group, since most of them were patients’ relatives. This could be a bias of the study since it could be expected that, under these conditions, the research subject could be more rigorous regarding preventive measures to avoid COVID-19, aiming to indirectly protect their family members.

Looking for possible differences in SARS-CoV-2 seroconversion among pediatric patients with ARDs, the underlying disease did not represent a significant risk factor itself. The same occurred regarding the use of immunosuppressive drugs, independent of the number and dose of medications taken. These observations demonstrate that, regardless of the immunosuppression mechanism, COVID-19 did not infect immunosuppressed children with ARDs more frequently, once more highlighting the differences between pediatric and adult rheumatic populations.^10^

Otherwise, Rituximab (RTX) inspires greater concern since this medication could reduce antibody production, increasing the risk of more severe infectious events.^30^ Two studies with adult populations analyzing the use of RTX and SARS-CoV-2 infection found an association between the use of this medication and higher morbidity and mortality.^9^^,^^31^ In the authors’ previous study with 14 rheumatic patients with symptomatic COVID-19, no serious involvement was seen, but none of them was in the use of RTX.^17^ After its publication, the authors had a single JDM adolescent patient who had received RTX and died from COVID-19 one month after its infusion (data not published). In the present study, the authors excluded patients who used RTX in the previous six months, aiming to avoid the interference of this medication in test results.

Analyzing the use of hydroxychloroquine, its use did not show efficacy in preventing COVID-19 since no differences were found between positive and negative patients related to the continuous use of this medication, as shown in previous studies.^8^^,^^17^

One important limitation of this study is the small sample of patients. The reason for this was the fact that the study was conducted during the second wave of COVID-19 in Brazil, which was much stronger than the first one, in 2020. As a result, only patients who really needed in-person appointments attended the present study's outpatient clinics. Even so, this sample of patients represents 12% of the total number of patients seen at the rheumatology unit (77/638). Also, despite the size of the study, it showed an important trend in the immunological behavior against COVID-19 in pediatric patients with ARDs.

Another limitation of the study is the size and selection of the control group, which was also linked to the second wave of COVID since during this period the patients could come to the outpatient clinics accompanied only by their guardians. So, the final control group was composed of 40% of health workers' relatives, which may have contributed to an increased intra-household contagion.

In conclusion, children and adolescents with ARDs were infected at the same rate as healthy ones. Furthermore, neither the underlying pathology nor its treatment seemed to interfere with the risk of contagion, which occurred mostly within their own residences. Larger and multicenter studies, including analyses of new variants, are needed to corroborate the present results.

## Authors' contributions

Juliana R Simon, Clovis A Silva and Lucia M A Campos participated in the conception, design of the work, acquisition, analysis, interpretation of data, and substantively revised the final text. Maria F B Pereira and Heloisa H Marques participated in the conception, design of the work, acquisition, and substantively revised the final text. Vera Bain participated in the conception, design of the work and acquisition of data. Juliana COA Ferreira, Neusa K Sakita, had actively participated in the acquisition of data. Adriana M Elias, Alexandre R Precioso, Sandra J F E Grisi, Ana PSF Barreto substantively revised the manuscript.

## Conflicts of interest

The authors declare no conflicts of interest.
